# Clinical Use of Tecovirimat (Tpoxx) for Treatment of Monkeypox Under an Investigational New Drug Protocol — United States, May–August 2022

**DOI:** 10.15585/mmwr.mm7137e1

**Published:** 2022-09-16

**Authors:** Kevin O’Laughlin, Farrell A. Tobolowsky, Riad Elmor, Rahsaan Overton, Siobhán M. O’Connor, Inger K. Damon, Brett W. Petersen, Agam K. Rao, Kevin Chatham-Stephens, Patricia Yu, Yon Yu, CDC Monkeypox Tecovirimat Data Abstraction Team, CDC Monkeypox Tecovirimat Data Abstraction Team, Sarah Ahmadi, Rachel Avery, Kathryn Bean, Leah Beavers, Kim Belanger Giguere, Joi Brownlee, Catherine Campbell, Maggie Cheng, Rachel Clinton, Taylor Coleman, Monique S. Davis, Marie Dubreus, Meryl Henry, Sujeith B. Lozoya, Jahnae Morgan, Kalimah Muhammad, Corinne M. Parker, Nigel Peters, Ellery Rybak, Andrew Schwenk, Jessica van Loben Sels, Max Veillard,

**Affiliations:** ^1^CDC Monkeypox Emergency Response Team; ^2^Booz Allen Hamilton Inc., McLean, Virginia.; CDC; CDC; CDC; CDC; CDC; CDC; CDC; CDC; CDC; CDC; CDC; CDC; CDC; CDC; CDC; CDC; CDC; CDC; CDC; CDC; CDC; CDC

Currently, no Food and Drug Administration (FDA)–approved treatments for human monkeypox are available. Tecovirimat (Tpoxx), however, is an antiviral drug that has demonstrated efficacy in animal studies and is FDA-approved for treating smallpox. Use of tecovirimat for treatment of monkeypox in the United States is permitted only through an FDA-regulated Expanded Access Investigational New Drug (EA-IND) mechanism. CDC holds a nonresearch EA-IND protocol that facilitates access to and use of tecovirimat for treatment of monkeypox.^§^ The protocol includes patient treatment and adverse event reporting forms to monitor safety and ensure intended clinical use in accordance with FDA EA-IND requirements. The current multinational monkeypox outbreak, first detected in a country where *Monkeypox virus* infection is not endemic in May 2022, has predominantly affected gay, bisexual, and other men who have sex with men (MSM) (*1*,*2*). To describe characteristics of persons treated with tecovirimat for *Monkeypox virus* infection, demographic and clinical data abstracted from available tecovirimat EA-IND treatment forms were analyzed. As of August 20, 2022, intake and outcome forms were available for 549 and 369 patients, respectively; 97.7% of patients were men, with a median age of 36.5 years. Among patients with available data, 38.8% were reported to be non-Hispanic White (White) persons, 99.8% were prescribed oral tecovirimat, and 93.1% were not hospitalized. Approximately one half of patients with *Monkeypox virus* infection who received tecovirimat were living with HIV infection. The median interval from initiation of tecovirimat to subjective improvement was 3 days and did not differ by HIV infection status. Adverse events were reported in 3.5% of patients; all but one adverse event were nonserious. These data support the continued access to and treatment with tecovirimat for patients with or at risk for severe disease in the ongoing monkeypox outbreak.

Tecovirimat^¶^ is an antiviral drug developed as a medical countermeasure to treat smallpox, a serious and life-threatening infection caused by *Variola virus*, of genus *Orthopoxvirus; Monkeypox virus* belongs to the same genus but typically causes less severe disease.** Global eradication of smallpox was declared by the World Health Assembly in 1980.^††^ Because opportunities to develop clinical trials in countries where *Monkeypox* v*irus* infection is considered endemic have been limited, the efficacy of tecovirimat to treat monkeypox has not been fully evaluated in humans. Instead, efficacy data that supported FDA approval of tecovirimat for smallpox were based on nonhuman primate and rabbit studies^§§^ (*3*); efficacy studies were also conducted in macaque monkeys and prairie dogs (*4*,*5*).

During May 2022, a multinational monkeypox virus outbreak (Clade II) was first reported, principally affecting MSM (*1*,*2*). Interim CDC guidance currently recommends that tecovirimat be considered in patients with severe disease, those at high risk for severe disease, or those with aberrant infections.^¶¶^ This report describes the available demographic and clinical characteristics, clinical indications for use, clinical outcomes, and adverse events reported among some of the first known recipients of tecovirimat treatment under the EA-IND protocol for *Monkeypox virus* infection in the United States.

During May 29–July 20, 2022, the EA-IND protocol required patient assessment forms at the start of treatment and once during three follow-up time points (assessment A: day 1–7, assessment B: day 8–14, and assessment C: posttreatment). Initially, the protocol's eligibility criteria included laboratory confirmation of *Monkeypox virus* or *Orthopoxvirus* infection, or a presumptive diagnosis based on clinical signs and symptoms. The revised EA-IND protocol (version 6, dated July 20, 2022) was amended as follows: 1) a patient may be eligible for tecovirimat based on clinical signs and symptoms and if there was an epidemiologic link to a case of or exposure to *Monkeypox virus*, 2) the number of follow-up visits was reduced to one during and one posttreatment, and 3) only reporting of serious adverse events using MedWatch forms was required.*** Data abstracted from patient intake forms included demographic characteristics, orthopoxvirus vaccination status, immune status,^†††^ laboratory test result, clinical signs and symptoms, reason for tecovirimat administration (i.e., lesions in sensitive anatomic areas, at risk for severe disease, and pain), formulation at the start of treatment (i.e., intravenous or oral), and number of days from symptom onset to administration of the first dose. Data from clinical outcome forms included whether the patient was hospitalized, number of days from initiation of treatment to subjective improvement, recovery status (i.e., recovered with or without sequelae or not yet recovered), and adverse events during and after treatment. The EA-IND protocol was reviewed and approved by CDC’s Institutional Review Board, reviewed and authorized by FDA, and conducted consistent with applicable federal law and CDC policy.^§§§^ Analyses were conducted using SAS software (version 9.4; SAS Institute). Difference in time to subjective improvement between HIV-positive and patients without HIV-positive status documented were compared using a 2-sided Wilcoxon rank-sum test; p<0.05 was considered statistically significant.

CDC abstracted data from patient intake forms for 549 persons with confirmed or suspected monkeypox who were prescribed tecovirimat therapy by August 20, 2022, and outcome forms for 369 patients. Data from both intake and outcome forms were available for 174 of these patients. Among 527 patients with intake forms and available data, 515 (97.7%) were male (Table 1). The median age was 36.5 years (IQR = 31.4–43.9 years). Among 464 patients with race and ethnicity data, 180 (38.8%) were White persons, 161 (34.7%) were Hispanic or Latino persons, and 83 (17.9%) were non-Hispanic Black or African American persons. Among 359 patients with available data, approximately two thirds (232, 64.6%) of tecovirimat recipients had lesions affecting <10% of their body; 17 (4.7%) had lesions affecting 75%–100% of their body. Among 529 patients with available data on number of lesions, 299 (56.5%) reported 10–100 lesions at the start of tecovirimat; 210 (39.7%) had fewer than 10 lesions, and 20 (3.8%) had more than 100 lesions. The presence of lesions in anatomic areas that might result in serious sequelae was reported on 191 (79.6%) of the 240 revised intake forms with available data. The most frequently reported underlying medical condition affecting immune status was HIV infection (254, 46.3%); viral load and CD4 count were not reported. Among 495 persons with available data on route of administration, the oral formulation of tecovirimat was prescribed to 494 (99.8%) at the start of therapy. Median interval from symptom onset to receipt of first tecovirimat dose was 7 days (IQR = 5–10 days) (Figure). Among 260 persons with revised intake forms, 124 (47.7%) had laboratory-confirmed *Orthopoxvirus* infection when tecovirimat treatment commenced.

**TABLE 1:** Demographic and clinical characteristics abstracted from intake forms of patients with *Monkeypox virus* infection who received tecovirimat (Tpoxx) under the Food and Drug Administration–regulated Expanded Access Investigational New Drug protocol (N = 549) — United States, May–August 2022

Characteristic (no. missing, unknown, or not specified)	No. (%)
Sex* (22)	
Male	515 (97.7)
Female	12 (2.3)
**Age group, yrs (22)**	
0–18	5 (0.9)
19–64	518 (98.3)
≥65	4 (0.8)
Median (IQR)	36.5 (31.4–43.9)
**Race and ethnicity (85)**	
White, non-Hispanic	180 (38.8)
Hispanic or Latino	161 (34.7)
Black or African American, non-Hispanic	83 (17.9)
Asian, non-Hispanic	13 (2.8)
Other, non-Hispanic	11 (2.4)
Unknown race, non-Hispanic	10 (2.2)
Multiple races, non-Hispanic	6 (1.3)
**HHS region^†^**	
2	141 (25.7)
3	68 (12.4)
4	88 (16.0)
5	36 (6.6)
9	153 (27.9)
Other regions	63 (11.5)
**Lifetime history of vaccination against monkeypox or smallpox** ^§^	
No monkeypox or smallpox vaccination documented	488 (88.9)
JYNNEOS	52 (9.5)
Previous monkeypox or smallpox vaccination reported, but vaccine product unknown	8 (1.5)
ACAM2000	1 (0.2)
**Percentage of body affected (190)**	
<10	232 (64.6)
10–24	60 (16.7)
25–49	28 (7.8)
50–74	22 (6.1)
75–100	17 (4.7)
Median (IQR)	5 (1–10)
**No. of lesions at start of treatment (20)**	
<10	210 (39.7)
10–100	299 (56.5)
>100	20 (3.8)
**Clinical indication for treatment**^¶^ **(not mutually exclusive) (309)**	
Lesions in anatomic areas that might result in serious sequelae**	191 (79.6)
At risk for severe disease	74 (30.8)
Pain	121 (50.4)
**Signs and symptoms documented at start of treatment (not mutually exclusive)**	
Rash	460 (83.8)
Fever	194 (35.3)
Rectal pain	108 (19.7)
Lymphadenopathy	74 (13.5)
Headache	46 (8.4)
Malaise	35 (6.4)
**Immune status**	
HIV-positive	254 (46.3)
Other immunocompromising condition**^††^**	7 (1.3)
No immunocompromising condition reported	288 (52.5)
**Route of administration (54)**	
Oral	494 (99.8)
Intravenous	1 (0.2)
**Days from exposure to onset of first symptoms (319)**	
Median (IQR)	7.0 (4–9)
**Days from onset of first symptoms to first dose (105)**	
Median (IQR)	7.0 (5–10)

**Abbreviation:** HHS = U.S. Department of Health and Human Services.

* The initial intake form captured sex without any other qualification, and the revised version of the form captured sex as “sex assigned at birth.” These two variables were combined to form the variable “sex”; therefore, some patients might have been misclassified.

^†^ HHS region 2 = New Jersey and New York; region 3 = District of Columbia, Maryland, Pennsylvania, and Virginia; region 4 = Alabama, Florida, Georgia, North Carolina, South Carolina, and Tennessee; region 5 = Illinois, Indiana, Michigan, Minnesota, Ohio, and Wisconsin; region 9 = Arizona, California, Hawaii, and Nevada; other regions combined = HHS region 1 (Connecticut, Massachusetts, and Rhode Island), HHS Region 6 (Arkansas, Louisiana, and Texas), HHS region 7 (Iowa, Kansas, Missouri, and Nebraska), HHS region 8 (Colorado), and HHS region 10 (Idaho, Oregon, and Washington).

^§^ Vaccination status was assessed by medical record and patient questionnaire, not by physical exam, so those unaware of vaccination status, even those with a scar from Dryvax, might have been missed.

^¶^ Clinical indication was only captured on the revised intake form; therefore, the denominator is reduced (240 revised intake forms with nonmissing data).

** Lesions in anatomic areas that might result in serious sequelae (e.g., eye, genitals, and oral mucosa) were not described in the form but reported based on the treating clinician’s assessment based on the phrase “lesions in sensitive anatomical areas.”

^††^ Includes receipt of immunosuppressive therapies and the following categories collected from the investigational new drug protocol paperwork: leukemia, lymphoma, bone marrow or organ transplant, congenital immune defects, or other cancers.

**FIGURE:**
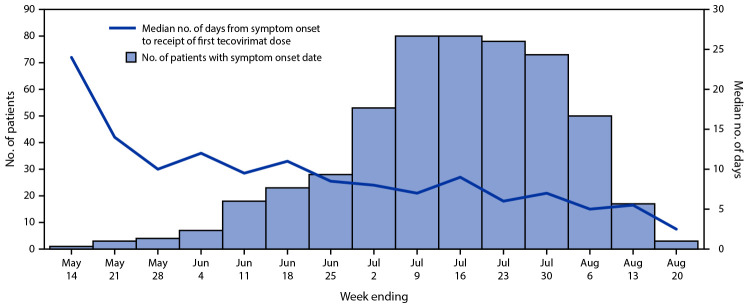
Interval from symptom onset to receipt of first tecovirimat (Tpoxx) dose* among symptomatic patients with *Monkeypox virus* infection treated under the Food and Drug Administration–regulated Expanded Access Investigational New Drug protocol (N = 518)^†^ — United States, May–August 2022 * Calculated from nonmissing values (444). ^†^ Overall, 31 patients with missing symptom onset date were excluded.

Among 369 patients with outcome forms, data on hospitalization status was available for 331; among these, 23 (6.9%) were hospitalized after symptom onset (Table 2), and the median duration of hospitalization was 4 days (IQR = 1–5 days). Among 255 patients with available data, the median time to subjective improvement after starting treatment was 3 days (IQR = 2–4 days).^¶¶¶^ Among 317 patients with available outcome information, 230 (72.6%) recovered with or without sequelae**** by or before completion of the posttreatment assessment; 87 (27.4%) patients were reported by clinicians to be not yet recovered, 78 of whom had not yet completed the standard 14-day tecovirimat treatment course. Adverse events were reported for 12 (3.5%) of 340 patients with information on adverse events; these included headache (three), nausea (two), visual disturbance (two), weakness (two), and hospitalization for psychiatric reasons (one).^††††^ At the time of the posttreatment follow-up visit, three (2.2%) of 137 persons with information available had developed new lesions compared with 25 (13.1%) who had developed new lesions during the first week of treatment. Most (119, 89.5%) patients reported that all lesions were crusted and healing with a new layer of skin under the scab following treatment. Among 174 patients with available data, the interval to subjective improvement did not differ between HIV-positive persons (42; median = 3 days) and persons without documentation of HIV positive status (64; median = 3 days) with available data (p = 0.83).

**TABLE 2:** Clinical outcomes abstracted from outcome forms of patients with *Monkeypox virus* infection who received tecovirimat (Tpoxx) under the Food and Drug Administration–regulated Expanded Access Investigational New Drug protocol (N = 369) — United States, May–August 2022

Outcome (no. unknown or missing)	No. (%)	
**Hospitalized (38)**		
Yes*	23 (6.9)	
Intensive care unit*	2 (0.6)	
No	308 (93.1)	
**Outcome^†^ (52)**		
Recovered without sequelae	189 (59.6)	
Recovered with sequelae	41 (12.9)	
Not yet recovered	87 (27.4)	
**Days to subjective improvement**^§^ **(114)**		
Median, days (IQR)	3.0 (2–4)	
**Adverse event**^¶^ **(29)**		
Yes	12 (3.5)	
No	328 (96.5)	
Median no. of days to follow up after treatment initiation (IQR)**		
During treatment: assessment A (day 1–7)	6 (4–7)	
During treatment: assessment B (day 8–14)	10 (8–13)	
Posttreatment: assessment C	21 (20–23)	
**Assessment A (day 1–7) (156)**	213 (57.7)	
**New lesions (22)**		
Yes	25 (13.1)	
No	166 (86.9)	
**All lesions crusted and healed with new layer of skin (59)**		
Yes	49 (31.8)	
No	105 (68.2)	
**Assessment B (day 8–14) (187)**	182 (49.3)	
**New lesions (19)**		
Yes	22 (13.5)	
No	141 (86.5)	
**All lesions crusted and healed with new layer of skin (25)**		
Yes		78 (49.7)
No		79 (50.3)
**Assessment C (posttreatment) (225)**		144 (39.0)
**New lesions (7)**		
Yes		3 (2.2)
No		134 (97.8)
**All lesions crusted and healed with new layer of skin (11)**		
Yes		119 (89.5)
No		14 (10.5)

* Hospitalized at any time after symptom onset. Among 23 patients hospitalized, two patients were admitted to the intensive care unit.

^†^At latest follow-up visit, which might have been during treatment or posttreatment. Recovery status was defined by clinical judgment of the treating provider.

^§^ Time to first observed (including patient-reported) improvement.

^¶^ All adverse events included headache (three), nausea (two), visual disturbance (two), weakness (two), vomiting (one), asymptomatic elevated liver function tests (one), hospitalization for psychiatric reasons (one), rash (one), hives (one), numbness (one), fatigue (one), and dizziness (one).

** Nonmissing data.

## Discussion

The data in this report support the continued availability of tecovirimat in the current monkeypox outbreak for U.S. patients with laboratory-confirmed or clinically diagnosed monkeypox. Initial findings indicate that tecovirimat is likely well tolerated; among reported adverse events, most were not serious, and it is not known whether tecovirimat caused the adverse events reported. The preliminary safety reporting with tecovirimat use under the EA-IND is consistent with data from the healthy human tecovirimat safety studies (SIGA-246–001).^§§§§^ Two other investigational treatments for orthopoxviruses, cidofovir and brincidofovir, have demonstrated substantial toxicity with limited efficacy data (*6*).

For patients treated under the EA-IND protocol and included in this report, the median time to subjective improvement was 3 days after receiving tecovirimat. However, no control group was available for comparison; therefore, no conclusions can be drawn regarding the effectiveness of tecovirimat to treat monkeypox based on these data. Time to improvement did not differ significantly with HIV infection status. A report from Nigeria suggested that HIV-positive patients might have prolonged illness; however, illness severity could be affected by HIV viral suppression, which was not reported in the current evaluation (*7*). Three of 137 patients (2.2%) with posttreatment follow-up and available data developed new lesions after completing treatment. A retrospective study in the United Kingdom reported one patient treated with tecovirimat had a shorter duration of illness compared with six patients (*8*), and another report of a small U.S. cohort treated with tecovirimat (also under the EA-IND) demonstrated complete resolution of lesions by day 21 in 23 (92%) of 25 patients (*9*).

This report illustrated that many patients were prescribed tecovirimat for lesions in anatomic areas that might result in serious sequelae, and nearly all received tecovirimat as outpatients, suggesting that severe disease was uncommon. The demographic characteristics of patients who received tecovirimat are similar to those with monkeypox: as described recently by CDC: the first 3,000 reported monkeypox infections in the United States occurred almost exclusively (99%) in men, with a median age of 35 years; approximately 40% were in White persons; and 41% of patients were living with HIV infection (*1*). Approximately one half of patients described in the EA-IND data did not have laboratory confirmation of *Monkeypox* *virus* infection and received tecovirimat empirically; because it is currently not known which patients benefit most from tecovirimat treatment, clinical judgment is important. Although this report could not evaluate efficacy, clinicians are encouraged to continue following CDC guidelines for tecovirimat use in patients with severe disease or at risk for severe disease. Because there is the potential for false-positive test results, tecovirimat should be considered only in those with a high pretest probability of being infected with *Monkeypox virus* to avoid unnecessary treatment or implementation of other public health measures (*10*). Inappropriate uses could potentially lead to resistance (*3*). Continued prescribing guidance updates on administering tecovirimat to those who would benefit the most from its use will be crucial as more is learned about effectiveness, viral resistance, and adverse events.

The findings in this report are subject to at least six limitations. First, only patients whose EA-IND forms were submitted to CDC were included in this report, representing a fraction of those treated to date^¶¶¶¶^; this limitation could lead to convenience sample bias that might not be representative of all patients treated with tecovirimat. Second, to lessen the regulatory burden on prescribers, the EA-IND forms were streamlined during the data collection process, leading to inconsistent variable collection. Third, some variables were collected as free text; therefore, absent data might not necessarily indicate absence of conditions. Fourth, the profile of patients might differ across assessment time points; for example, those with worse initial symptoms might have been more likely to receive follow-up assessments, making true time to resolution or improvement difficult to ascertain. Fifth, it is not known whether the outcomes described for patients who received tecovirimat differ from those of patients who do not receive tecovirimat because no control group was included. Finally, CD4 count and viral load, markers of unsuppressed HIV infection, were not collected, limiting the evaluation of treatment outcomes for persons living with HIV infection.

Ongoing monitoring is essential to assess the safety of tecovirimat in patients with *Monkeypox virus* infection under the EA-IND during the current monkeypox outbreak. CDC is continuing to review additional data as they become available. Currently, there are no human data demonstrating the efficacy of tecovirimat, and clinical trials are necessary to elucidate clinical efficacy in patients with *Monkeypox virus* infection, indications for treatment, and ideal duration of treatment.

**Summary**

**What is already known about this topic?**

Tecovirimat (Tpoxx) was approved by the Food and Drug Administration for treatment of smallpox based on data obtained from animal models; there are no safety or efficacy data regarding its use in patients with *Monkeypox virus* infection.

**What is added by this report?**

Among 549 patients with *Monkeypox virus* infection treated with tecovirimat under an Expanded Access Investigational New Drug protocol, 99.8% received it orally as an outpatient. Among 369 patients, few adverse events were reported.

**What are the implications for public health practice?**

Tecovirimat is generally well tolerated, and these data support continued access to treatment with tecovirimat during the current monkeypox outbreak.

## Footnotes

* These authors contributed equally as first authors.

† These authors contributed equally as senior authors.

§
https://www.cdc.gov/poxvirus/monkeypox/pdf/Tecovirimat-IND-Protocol-CDC-IRB.pdf

¶ Initially developed by the U.S. government, and, during later stages, in partnership with SIGA Technologies, Incorporated.

**
https://www.who.int/health-topics/monkeypox

††
https://www.cdc.gov/smallpox/history/history.html

§§ Animals were challenged, respectively, with *Monkeypox* *virus* and *Rabbitpox* *virus.*

¶¶ Aberrant infections involve accidental implantation in eyes, mouth, or other sensitive anatomic areas where *Monkeypox virus* infection might constitute a special hazard (e.g., the genitals or anus). It was previously thought that *Monkeypox virus* infections rarely affected these regions, so the term “aberrant” was used early in the 2022 multinational outbreak. The revised intake form used the phrase “sensitive anatomic areas” as shorthand for the preceding definition. The preferred phrase is “anatomic areas which might result in serious sequelae”. https://www.cdc.gov/poxvirus/monkeypox/clinicians/Tecovirimat.html

*** Serious adverse event (AE) defined as death, life-threatening event, inpatient hospitalization or prolongation of existing hospitalization, persistent or significant incapacity or substantial disruption of the ability to conduct normal life functions, congenital anomaly/birth defect, or an important medical event that based on appropriate medical judgment might jeopardize the patient and might require medical or surgical intervention to prevent one of the aforementioned outcomes.

††† Includes receipt of immunosuppressive therapies and the following categories collected from the investigational new drug protocol paperwork: leukemia, lymphoma, bone marrow or organ transplant, congenital immune defects, or other cancers.

§§§ 45 C.F.R. part 46, 21 C.F.R. part 56; 42 U.S.C. Sect. 241(d); 5 U.S.C. Sect. 552a; 44 U.S.C. Sect. 3501 et seq.

¶¶¶ Time to first observed (including patient-reported) improvement.

**** Per clinical judgment of the treating provider.

†††† All adverse events included headache (three), nausea (two), visual disturbance (two), weakness (two), vomiting (one), asymptomatic elevated liver function tests (one), depression with suicidality (one), rash (one), hives (one), numbness (one), fatigue (one), and dizziness (one).

§§§§
https://www.clinicaltrials.gov/ct2/show/NCT02474589

¶¶¶¶ In comparison with the monkeypox cases reported in the United States (15,433 cases through August 20, 2022).https://www.cdc.gov/poxvirus/monkeypox/response/2022/mpx-trends.html
